# Association between estimated glucose disposal rate and metabolic syndrome: a cross-sectional analysis of the National Health and Nutrition Examination Survey

**DOI:** 10.3389/fnut.2025.1544582

**Published:** 2025-03-24

**Authors:** Kaide Xia, Shuai Jin, Guifang Chen, Haiwang Zhang, Qiao Zhang

**Affiliations:** ^1^Guiyang Maternal and Child Health Care Hospital, Guiyang Children's Hospital, Guiyang, China; ^2^School of Biology and Engineering (School of Health Medicine Modern Industry), Guizhou Medical University, Guiyang, China; ^3^Department of Pharmacy, The People's Hospital of Guiyang City Yunyan District, Guiyang, China; ^4^Department of Neurosurgery, Guizhou Provincial People's Hospital, Guiyang, China; ^5^Department of Hospital Management, The Second People's Hospital of Guiyang, Guiyang, China

**Keywords:** metabolic syndrome, NHANES, eGDR, logistic regression, RCS

## Abstract

Metabolic syndrome (MS) is a complex metabolic disorder that is often closely associated with the development of chronic diseases such as cardiovascular disease and diabetes. This study aimed to explore the relationship between estimated glucose metabolic rate (eGDR) and MS. The correlation between eGDR levels and the prevalence of metabolic syndrome was analyzed here based on data from the National Health and Nutrition Examination Survey from 2005 to 2020. The study sample consisted of 63,131 adult participants, and the results showed that lower eGDR levels were significantly associated with a higher prevalence of metabolic syndrome. Further regression analyses showed that eGDR acted as a protective factor and that the risk of MS significantly decreased as its level increased. Subgroup analyses showed that this trend held across gender, age, and BMI categories, and that the protective effect of eGDR was weaker in the higher BMI group. Based on the nonlinear relationship between subjects’ eGDR levels and MS prevalence, RCS analyses further confirmed a significant correlation between lower eGDR levels and increased risk of MS. In conclusion, the present study suggests that eGDR levels could serve as a potential biomarker for predicting metabolic syndrome, providing new perspectives for early screening and intervention of MS.

## 1 Introduction

Metabolic syndrome (MS) is a complex metabolic disorder characterized by central obesity, atherosclerotic dyslipidemia, hypertension, insulin resistance, prothrombotic and proinflammatory states, among other features (1, 2). Approximately 20 to 25% of adults worldwide are affected by MS (3, 4). According to data from the National Health and Nutrition Examination Survey (NHANES), about one-third of adults were diagnosed with MS between 1988 and 2010 (5). In China, the prevalence of MS among adults was 24.2% in 2018, with a higher rate of 24.6% in males compared to 23.8% in females (6). Early prediction and diagnosis of MS are crucial for timely intervention, which can not only effectively reduce health risks associated with MS but also alleviate the burden on the healthcare system (7, 8).

Currently, the diagnostic criteria for MS include waist circumference, blood glucose, blood pressure, triglycerides, and high-density lipoprotein cholesterol (HDL-C) (2). In addition, composite indices based on combinations of different metrics, such as the glucose–glucose index (TyG) and TyG-waist circumference, have demonstrated better results in the diagnosis and prognosis of MS (9, 10). Therefore, establishing and evaluating the impact of various physiological and biochemical indicators on the prevalence of MS is of significant theoretical and practical importance.

Williams et al. developed an index for assessing insulin resistance, known as the estimated glucose disposal rate (eGDR), based on the euglycaemic hyperinsulinaemic clamp (11). The adjusted eGDR formula proposed by Epstein and colleagues has since been more widely applied (12). The index integrates clinical data such as blood pressure, hemoglobin A1c levels, and waist circumference, and is simple to calculate. Although eGDR was originally developed to assess insulin resistance in individuals with type 1 diabetes, it has since been recognized as a general biomarker for metabolic syndrome and is closely associated with various diseases, including cardiovascular diseases and diabetes (13–15). However, research on the relationship between eGDR levels and the prevalence of MS is still insufficient. Therefore, this study will conduct a retrospective analysis using data from the National Health and Nutrition Examination Survey conducted between 2005 and 2020, focusing on individuals with MS. The primary objective is to systematically explore the potential of eGDR levels as a predictive marker for MS prevalence, providing a scientific foundation for its potential role in the prevention and treatment of MS.

## 2 Materials and methods

### 2.1 Sample sources

The data used in this study were derived from the National Health and Nutrition Examination Survey, a major program of the National Center for Health Statistics that provides national health and nutrition statistics. The NHANES aims to assess the health and nutritional status of adults and children in the United States, utilizing a combination of interviews and physical examinations (16). This study collected data on eGDR levels and metabolic syndrome across three survey cycles: 2005–2006, 2007–2008, and 2009–2010. The NHANES protocol has been approved by the NCHS Research Ethics Review Board and by all participants, eliminating the need for additional institutional review board approval. The primary objective of this study was to investigate the relationship between eGDR levels and the prevalence of metabolic syndrome.

### 2.2 About eGDR levels

The eGDR is a clinical indicator used to assess an individual’s insulin sensitivity and the degree of insulin resistance, reflecting the effectiveness of insulin metabolism. The calculation of eGDR levels incorporates easily accessible clinical parameters, such as glycated hemoglobin (HbA1c), blood pressure status (hypertension or normal blood pressure), and waist circumference. The formula for calculating eGDR is as follows: eGDR (mg/kg/min) = 21.158 - (0.09 × Waist circumference in cm) - (3.407 × Hypertension, 1 = Yes, 0 = No) - (0.551 × HbA1c%) (12). A decrease in eGDR levels typically indicates an increase in insulin resistance, while higher eGDR values are associated with better insulin sensitivity.

### 2.3 Definition of metabolic syndrome

MS is a pathological condition characterized by a cluster of metabolic abnormalities that are closely associated with an increased risk of chronic diseases such as cardiovascular disease and type 2 diabetes. It consists of a series of interrelated metabolic disturbances, including abdominal obesity, hyperglycemia, dyslipidemia, and hypertension. In this study, the diagnosis of MS was based on the modified criteria of the National Cholesterol Education Program Adult Treatment Panel III (NCEP: ATP III). MS is diagnosed if three or more of the following five conditions are met: (1) Abdominal Obesity: Waist circumference ≥ 40 inches (102 cm) in men; ≥ 35 inches (88 cm) in women. (2) Triglycerides: ≥ 150 mg/dL (1.7 mmol/L). (3) Low HDL Cholesterol (HDL-C): < 40 mg/dL (1.0 mmol/L) in men; < 50 mg/dL (1.3 mmol/L) in women. (4) Elevated Blood Pressure: Systolic blood pressure ≥ 130 mmHg or diastolic blood pressure ≥ 85 mmHg, or currently receiving treatment for hypertension. (5) Elevated Fasting Glucose: ≥ 100 mg/dL (5.6 mmol/L), or currently receiving treatment for diabetes. The final outcome of this study was the presence or absence of MS.

### 2.4 Selection and processing of covariates

In addition to eGDR levels, this study incorporated several demographic variables, including age, sex, race, marital status, education level, poverty status, physical activity, body mass index (BMI), and alcohol and tobacco use. Race was categorized into four groups: White, Mexican American, Black individuals, and Other. Marital status was classified as married, divorced, widowed, separated, never married, or living with a partner. Education level was categorized as high school or below, high school or equivalent, and above high school. Past medical history data included cancer, comorbidities, hypertension, and diabetes, all of which were treated as dichotomous variables. Additionally, laboratory variables such as triglycerides (TG, mmol/L), total cholesterol (TC, mmol/L), high-density lipoprotein cholesterol (HDL-C, mmol/L), and low-density lipoprotein cholesterol (LDL-C, mmol/L) were also collected. These covariates will be adjusted for in the statistical analysis.

### 2.5 Statistical methods

Data analysis was conducted using complex sampling weights recommended by the Centers for Disease Control and Prevention. Following the methodology outlined on the NHANES website, we combined the sample weights from the 2005–2006, 2007–2008, and 2009–2010 cycles. eGDR levels were categorized into tertiles based on prior literature. Continuous variables with a normal distribution were expressed as means (SD), while skewed continuous variables were reported as medians (IQR) and analyzed using the T-test or rank-sum test. Categorical variables were presented as proportions and compared between groups using the χ^2^ test.

To examine the relationship between eGDR levels and the prevalence of MS, we performed both univariate and multivariate logistic regression analyses. In Model 1, only the relationship between eGDR tertiles and MS prevalence was examined. Model 2 adjusted for age, sex, and race/ethnicity, and Model 3 further adjusted for all covariates. To explore the nonlinear relationship between eGDR levels and MS, we applied the restricted cubic spline (RCS) method. All statistical analyses and visualizations were conducted using R software (https://www.r-project.org/, Version 4.3.1), with a significance level set at *p* < 0.05.

## 3 Results

### 3.1 Baseline demographic and clinical characterization

**Figure 1:**
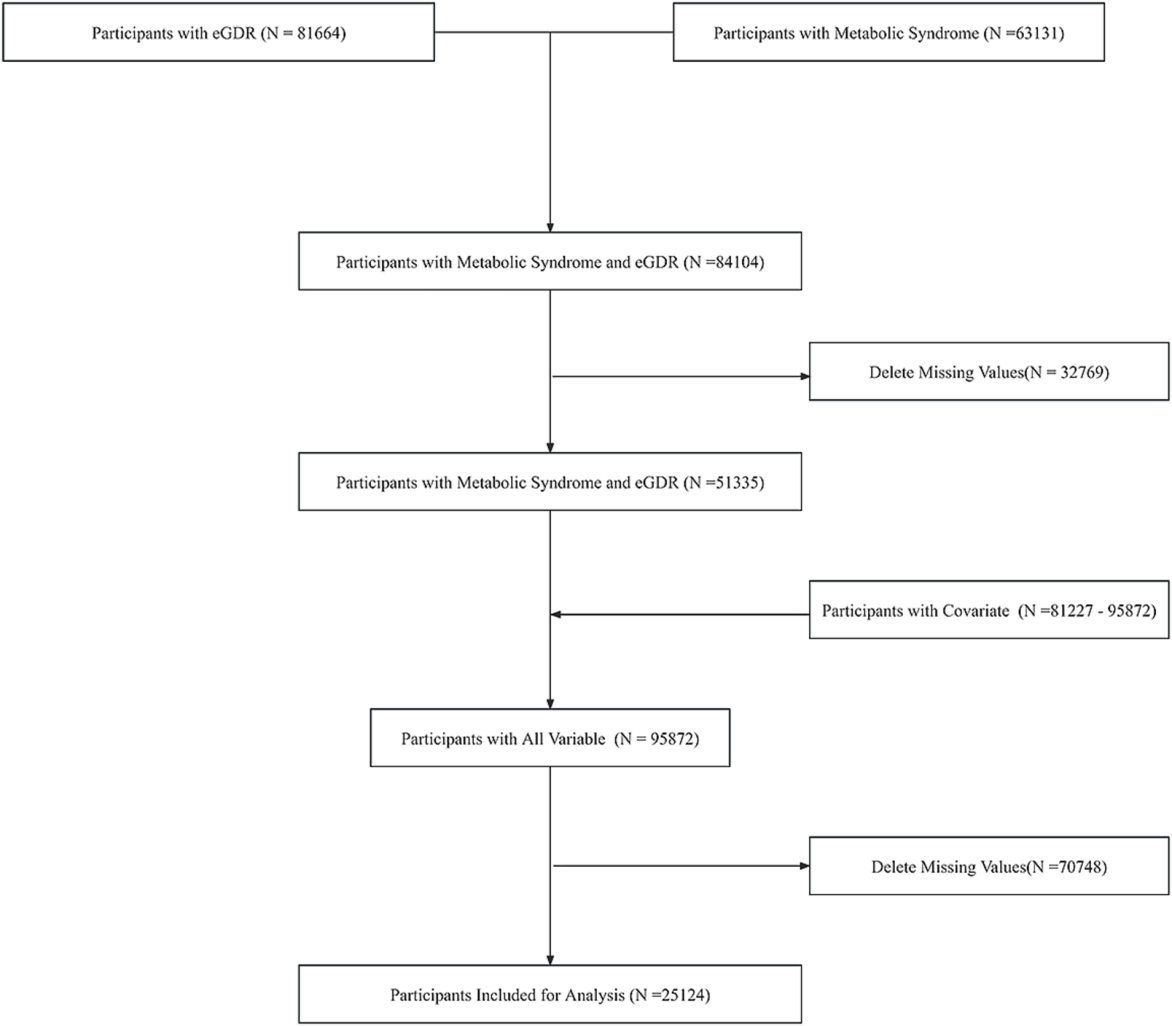
Sample screening flowchart.

Our study initially extracted data from the NHANES database, comprising 63,131 individuals with metabolic syndrome information and 81,664 individuals with eGDR data. Following data integration, a combined cohort of 84,104 participants was established. After excluding 32,769 individuals with missing values, the remaining sample consisted of 51,335 participants. The covariates exhibited varying sample sizes, ranging from 81,227 to 95,872 across different parameters. Subsequent integration of covariate information yielded a comprehensive dataset of 95,872 individuals. Following the exclusion of 70,748 participants with any missing values, our final analytical cohort comprised 25,124 individuals. Quality control measures confirmed the absence of outliers in the final dataset. Weighted to represent 138,013,430 participants. The screening flowchart is shown in Figure 1.

Based on eGDR levels, all samples were categorized into three groups by tertiles: Q1 (eGDR range: −4.232 to 6.42), Q2 (6.42 to 9.558), and Q3 (9.558 to 13.106). Table 1 demonstrates the between-group differences in baseline demographic characteristics and eGDR level subgroups. The study population was 52% male and 48% female, with a mean age of 45.7 years. Higher proportions were White individuals, married, had a high school education or higher, had never smoked, and were light drinkers. The majority of study participants did not have diabetes or cancer, 34% had hypertension, and 21.26% had metabolic syndrome.

**Table 1:** Differential analysis of baseline characteristics in weighted eGDR quartiles.

Characters	Total	Q1	Q2	Q3	*P* Value
Sex, %					< 0.001
Male	51.84	59.13	54.58	43.47	
Female	48.16	40.87	45.42	56.53	
Age, mean (SD)	45.74 (0.24)	54.40 (0.28)	46.54 (0.28)	38.11 (0.28)	< 0.001
BMI, mean (SD)	28.72 (0.08)	33.39 (0.12)	29.92 (0.08)	23.88 (0.05)	< 0.001
Race/Ethnicity, %					< 0.001
White	70.62	71.18	70.49	70.29	
Mexican	7.58	6.42	9.12	7.06	
Black individuals	9.63	12.42	8.77	8.22	
Other	12.17	9.98	11.62	14.44	
Marital status, %					< 0.001
Married	57.16	62.11	59.59	50.95	
Living with partner	7.45	4.89	7.91	9.06	
Separated	1.92	2.12	1.89	1.79	
Divorced	9.00	11.28	9.07	7.12	
Widowed	5.45	8.96	5.52	2.61	
Never married	19.02	10.65	16.01	28.47	
Education, %					< 0.001
Under high school	11.82	13.52	12.12	10.19	
High school or equivalent	22.67	25.79	24.11	18.85	
Above high school	65.51	60.69	63.77	70.97	
Poverty, median (IQR)	3.34 (1.69,5.00)	3.23 (1.65,5.00)	3.29 (1.74,5.00)	3.45 (1.69,5.00)	0.46
Physical Activity, median (IQR)	1920.00 (720.00,5300.00)	1680.00 (567.00,4800.00)	1920.00 (720.00,5760.00)	2280.00 (840.00,5760.00)	< 0.001
Smoking, %					< 0.001
Never	55.62	50.60	54.42	60.72	
Former	24.79	33.28	24.28	18.52	
Now	19.59	16.12	21.30	20.76	
Alcohol intake, %					< 0.001
Never	8.93	9.42	8.50	8.94	
Former	10.06	13.56	10.70	6.69	
Mild	38.85	41.22	39.09	36.75	
Moderate	18.97	16.33	18.06	21.92	
Heavy	23.19	19.47	23.66	25.70	
Diabetes, %					< 0.001
No	91.59	78.11	94.77	99.35	
Yes	8.41	21.89	5.23	0.65	
Hypertension, %					< 0.001
No	65.80	7.68	78.67	100.00	
Yes	34.20	92.32	21.33	0.00	
Cancer, %					< 0.001
No	90.78	86.27	91.13	94.04	
Yes	9.22	13.73	8.87	5.96	
Metabolic syndrome, %					< 0.001
No	78.74	48.32	83.72	98.29	
Yes	21.26	51.68	16.28	1.71	

In the eGDR tertile subgroups, concerning gender, the proportion of males was higher in the Q1 and Q2 groups, while the proportion of females was higher in the Q3 group. Age and BMI tended to decrease as the eGDR quartile increased. In terms of race, the Q3 group had a higher percentage of other races, while the Q1 group had a higher percentage of Black individuals. In terms of marital status, the married group had the largest percentage in the Q1 group, while those who had never been married had the highest percentage in the Q3 group. The group with higher educational level has the largest percentage in group Q3. Individuals with higher poverty index and more physical activity tended to be located in the higher eGDR quartile groups. Smoking prevalence declines with increasing eGDR quartiles, while the proportion of moderate and higher level drinkers increases with higher eGDR. The proportion of people with diabetes, hypertension and cancer decreased with higher eGDR quartiles. The prevalence of metabolic syndrome followed a similar trend, with a higher proportion in the Q3 group not suffering from MS.

### 3.2 Relationship between eGDR level and MS prevalence

The logistic regression analysis results showed that eGDR was negatively associated with MS prevalence in Model 1, Model 2 and Model 3. Both in the model containing only eGDR and in the logistic regression model with different covariates, eGDR was found to be a protective factor for MS prevalence. According to the results of eGDR tertile analysis, in model 1, with Q1 as the control group, both Q2 and Q3 groups showed a protective effect. After covariate correction, the results in models 2 and 3 were consistent with model 1. The *p*-values of the trend tests were all less than 0.01, indicating that there was indeed a linear relationship between eGDR and MS prevalence after using eGDR as an ordered categorical variable (Table 2).

**Table 2:** Weighted ORs for the associations between eGDR and MS across three models.

Exposure	Model 1		Model 2		Model 3	
	OR (95%CI)	*P* value	OR (95%CI)	*P* value	OR (95%CI)	*P* value
eGDR (mg/kg/min)	0.56 (0.55, 0.57)	<0.001	0.56 (0.55, 0.58)	<0.001	0.48 (0.44, 0.52)	<0.001
Quartile of eGDR (mg/kg/min)						
Q1	Ref	Ref	Ref	Ref	Ref	Ref
Q2	0.18 (0.17, 0.20)	<0.001	0.19 (0.17, 0.20)	<0.001	0.35 (0.30, 0.42)	<0.001
Q3	0.02 (0.01, 0.02)	<0.001	0.02 (0.01, 0.02)	<0.001	0.07 (0.05, 0.09)	<0.001
P for trend		<0.001		<0.001		<0.001

Model 1: Add eDGR. Model 2: Add eDGR, Age, Sex, and Race/Ethnicity. Model 3: All the covariates. eGDR: Estimated glucose disposal rate; MS: Metabolic syndrome.

### 3.3 Subgroup analysis

Table 3 presents the results of the analysis of eGDR levels and their tertiles across different subgroups. The findings revealed a significant negative correlation between eGDR levels and MS prevalence in all covariate subgroups. Specifically, higher eGDR levels were associated with a lower prevalence of MS, indicating a protective effect. The odds ratios (ORs) were all less than 0.6 in every subgroup, except in the subgroup with higher BMI, suggesting that the protective effect of eGDR levels was significant in most subgroups. In the subgroup analyses by eGDR tertiles, using the Q1 group as a reference, the ORs for the Q2 group were all below 0.5 (*p* < 0.001), while the ORs for the Q3 group were all below 0.2 (p < 0.001). The higher the eGDR level, the more pronounced the protective effect, which was consistent with the observed negative correlation between eGDR levels and MS prevalence.

**Table 3:** Stratified logistic regression analysis.

Character	eGDR (mg/kg/min)		Quartile of eGDR (mg/kg/min)				
	OR (95%CI)	*P* value	Q1	Q2		Q3	
			OR (95%CI)	OR (95%CI)	*P* value	OR (95%CI)	*P* value
Sex							
Male	0.50 (0.44, 0.57)	<0.001	Ref	0.45 (0.34, 0.59)	<0.001	0.06 (0.03, 0.10)	<0.001
Female	0.46 (0.42, 0.52)	<0.001	Ref	0.28 (0.22, 0.36)	<0.001	0.06 (0.04, 0.09)	<0.001
Age							
= < 60	0.46 (0.42, 0.50)	<0.001	Ref	0.35 (0.28, 0.43)	<0.001	0.05 (0.03, 0.07)	<0.001
>60	0.44 (0.37, 0.52)	<0.001	Ref	0.29 (0.22, 0.40)	<0.001	0.11 (0.06, 0.20)	<0.001
BMI							
Normal	0.33 (0.28, 0.39)	<0.001	Ref	0.28 (0.20, 0.40)	<0.001	0.06 (0.03, 0.09)	<0.001
Low	0.27 (0.21, 0.35)	<0.001	Ref	0.21 (0.13, 0.34)	<0.001	0.02 (0.01, 0.06)	<0.001
High	0.74 (0.70, 0.78)	<0.001	Ref	0.43 (0.32, 0.58)	<0.001	0.18 (0.10, 0.31)	<0.001
Smoke							
Never	0.49 (0.44, 0.55)	<0.001	Ref	0.40 (0.30, 0.52)	<0.001	0.07 (0.04, 0.11)	<0.001
Former	0.48 (0.41, 0.55)	<0.001	Ref	0.31 (0.22, 0.42)	<0.001	0.05 (0.02, 0.10)	<0.001
Now	0.43 (0.37, 0.51)	<0.001	Ref	0.28 (0.18, 0.43)	<0.001	0.06 (0.03, 0.13)	<0.001
Alcohol							
Never	0.52 (0.44, 0.62)	<0.001	Ref	0.41 (0.28, 0.61)	<0.001	0.11 (0.05, 0.21)	<0.001
Former	0.50 (0.40, 0.62)	<0.001	Ref	0.29 (0.17, 0.49)	<0.001	0.07 (0.03, 0.16)	<0.001
Mild	0.47 (0.42, 0.53)	<0.001	Ref	0.37 (0.27, 0.52)	<0.001	0.05 (0.03, 0.10)	<0.001
Moderate	0.36 (0.30, 0.43)	<0.001	Ref	0.33 (0.20, 0.55)	<0.001	0.05 (0.02, 0.11)	<0.001
Heavy	0.55 (0.48, 0.64)	<0.001	Ref	0.34 (0.24, 0.46)	<0.001	0.08 (0.05, 0.13)	<0.001

### 3.4 RCS of eDGR and MS prevalence

**Figure 2:**
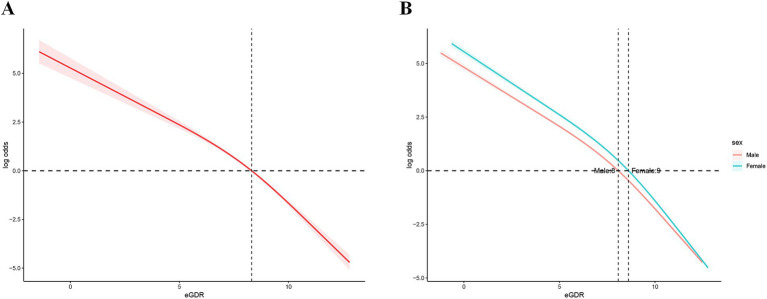
Overall and gender-specific RCS curves. RCS: restricted cubic spline.

The RCS analysis revealed a significant nonlinear association between eGDR levels and the log odds ratio of MS events (Figure 2A), which remained consistent across genders (Figure 2B). Specifically, the log probability of an MS event was significantly reduced with increasing eGDR levels, indicating that an increase in eGDR levels is effective in reducing the risk of an MS event. However, this relationship is nonlinear. At low eGDR levels (eGDR<8), the log odds ratio decreases more slowly, suggesting that low eGDR levels have a limited effect on reducing the risk of MS events. As eGDR levels increased further (≥8), the rate of decrease in the log odds ratio increased, indicating that the effect of eGDR on reducing the risk of MS events became more pronounced and stronger at higher eGDR levels. This non-linear change suggests that eGDR may have a stronger protective effect after some cut points.

In addition, we further verified the stability of the curve fit over the entire range of eGDR levels by confidence interval analysis. This means that the non-linear relationship between eGDR and risk of MS events is reliable and consistent across different eGDR levels. These results further highlight the potential role of eGDR as an important indicator in MS risk assessment, especially at high eGDR levels, which may have greater clinical significance.

## 4 Discussion

In this epidemiologic study, the US population was carefully screened to ensure it was representative of the study cohort, and a significant association was found between higher eGDR levels and a reduced prevalence of MS in a population without MS. This association remained significant even after adjusting for known risk factors for MS development. Furthermore, the negative correlation between eGDR levels and MS risk was consistent across stratified analyses of risk factors and socio-behavioral variables.

The metabolic syndrome contributes to the spread of diseases such as type 2 diabetes, coronary heart disease, stroke, and other disabilities (17, 18). A major driver of this phenomenon is the high degree of overlap between the core pathological features of the metabolic syndrome (e.g., insulin resistance, chronic low-grade inflammation, and abnormalities of lipid metabolism) and the pathogenesis of the aforementioned disorders (19, 20). This further increases the risk of coronary heart disease and stroke (21). In addition, chronic low-grade inflammation, an important feature of metabolic syndrome, leads to elevation of pro-inflammatory cytokines (e.g., TNF-*α*, IL-6), which induces systemic inflammatory responses and thus plays a key role in the course of many diseases (22, 23). Therefore, the level of eGDR levels may not only serve as a reliable marker of insulin resistance but may also indirectly reflect the chronic low-grade inflammation and abnormal lipid metabolism changes, thus providing a new perspective for early screening of metabolic syndrome and its related complications.

Despite the widespread health risks associated with MS, current diagnostic and management strategies remain limited. The internationally accepted diagnostic criteria for MS are mainly based on the definitions and recommendations of relevant international organizations and authoritative guidelines. For instance, the International Diabetes Federation uses a waist circumference-centered metabolic standard, combined with other metabolic risk factors (24). The National Cholesterol Education Program Adult Treatment Panel III incorporates a comprehensive set of indicators, including waist circumference, blood lipids, blood glucose, and blood pressure (1). Additionally, the World Health Organization emphasizes insulin resistance as a core factor, alongside indicators of blood glucose, blood pressure, and dyslipidemia (25). While these guidelines provide useful frameworks for identifying metabolic syndrome, they still fall short in disease prediction and individualized risk assessment. Existing criteria lack comprehensive and unified metrics for diagnosing MS. eGDR levels, as an insulin resistance-based biomarker, show promise in integrating the core pathological features of metabolic syndrome. Our study found that eGDR levels are not only significantly correlated with the prevalence of MS, but their stability in stratified analyses also suggests their potential application across different subgroups. Given that metabolic syndrome is a complex medical diagnosis involving multiple biomarkers, simple anthropometric definitions (such as waist circumference and blood pressure) have certain limitations for clinical application. In contrast, eGDR levels provide a more straightforward and acceptable alternative, offering an effective screening tool for large populations, particularly in resource-limited settings and high-risk groups (17).

As a marker of insulin resistance, eGDR levels exhibit accuracy comparable to the normoglycemic hyperinsulinemic clamp, making them suitable for clinical practice (26, 27). The normoglycemic hyperinsulinemic clamp, considered the gold standard for identifying and quantifying insulin resistance, is labor-intensive, time-consuming, and invasive, rendering it impractical for routine clinical use (28). Alternative methods, such as the homeostatic model assessment of insulin resistance, which relies on fasting insulin and glucose levels, have been proposed (29). However, simpler and more reliable alternatives are needed. Consequently, eGDR levels have been widely utilized in studies predicting vascular disease mortality (15, 30, 31), adverse nephropathic outcomes (22), adverse nephropathic outcomes (32), demonstrating excellent predictive efficacy. Similarly, eGDR levels have shown high sensitivity in predicting MS-related conditions, such as chronic diabetes mellitus (33) and the onset of type 1 diabetes (34). Notably, eGDR levels have demonstrated superior diagnostic performance for identifying metabolic syndrome in patients with type 1 diabetes, including children and adolescents (13, 35, 36), a finding consistent with the present study. Our study found that eGDR levels exhibited a protective effect across different racial groups, suggesting the potential for incorporating eGDR levels into routine diabetes care (33). However, it has also been shown that, in a population of type 1 diabetic patients from an urban clinic, lower eGDR levels in the Black individuals population were associated with diabetic complications (12). The inconsistency in these findings may be due to differences in the study samples. For example, genetic predispositions, lifestyle factors, or socioeconomic status in Black individuals populations may influence the relationship between eGDR levels and diabetic complications (37). Given the significant association between eGDR and MS risk, low eGDR levels could serve as an early indicator for identifying high-risk individuals, particularly those with existing metabolic risk factors. This can guide personalized interventions, such as lifestyle modifications and, where necessary, pharmacological treatment. Regular monitoring of eGDR levels could also facilitate the assessment of intervention efficacy and enable timely adjustments to treatment plans. However, despite eGDR’s significant potential in MS prediction, further research is needed to validate its standardization and application across diverse populations and clinical settings.

Although this study clarified the stable association between eGDR levels and the prevalence of MS, several potential limitations should be acknowledged. First, this was a cross-sectional study, which precludes the establishment of a causal relationship between eGDR levels and MS prevalence, limiting our ability to make causal inferences. Second, NHANES data are primarily based on U.S. populations, which may limit their applicability to other countries or regions. Third, NHANES data reflect health status at a single point in time, which prevents tracking of health changes or the effects of interventions over time. This represents a limitation in assessing the long-term progression of metabolic syndrome and fluctuations in eGDR levels. Fourth, a more detailed exploration of subgroup analysis is needed. For example, the weaker protective effect of eGDR in individuals with high BMI compared to other BMI groups warrants further investigation. Finally, NHANES did not consistently record whether participants received specific health interventions or treatments, such as glucose-lowering medications or insulin therapy, which may influence the relationship between eGDR levels and MS. In conclusion, while our findings provide valuable insights, they should be interpreted with caution. Further studies are needed to confirm the direction and strength of the relationship between eGDR levels and MS prevalence.

## 5 Conclusion

In conclusion, our findings suggest a significant association between higher eGDR levels and a lower prevalence of MS. However, further large-scale prospective studies are needed to validate these findings. Nonetheless, eGDR levels demonstrate important clinical utility as a valid predictor of MS prevalence and a potential therapeutic target.

## Data Availability

The raw data supporting the conclusions of this article will be made available by the authors, without undue reservation.
